# Riboflavin Responsive Mitochondrial Dysfunction in Neurodegenerative Diseases

**DOI:** 10.3390/jcm6050052

**Published:** 2017-05-05

**Authors:** Tamilarasan Udhayabanu, Andreea Manole, Mohan Rajeshwari, Perumal Varalakshmi, Henry Houlden, Balasubramaniem Ashokkumar

**Affiliations:** 1 Department of Genetic Engineering, School of Biotechnology, Madurai Kamaraj University, Madurai 625021, India; udhaya13banu@gmail.com (T.U.); ponderofbiotech@gmail.com (M.R.); 2 Department of Molecular Neuroscience and Neurogenetics Laboratory, UCL Institute of Neurology, Queen Square, London WC1N 3BG, UK; andreea.manole.13@ucl.ac.uk (A.M.); h.houlden@ucl.ac.uk (H.H.); 3 Department of Molecular Microbiology, School of Biotechnology, Madurai Kamaraj University, Madurai 625021, India; vara5277@gmail.com

**Keywords:** riboflavin, FAD, FMN, BVVLS, motor neuronopathy, mitochondrial dysfunction

## Abstract

Mitochondria are the repository for various metabolites involved in diverse energy-generating processes, like the TCA cycle, oxidative phosphorylation, and metabolism of amino acids, fatty acids, and nucleotides, which rely significantly on flavoenzymes, such as oxidases, reductases, and dehydrogenases. Flavoenzymes are functionally dependent on biologically active flavin adenine dinucleotide (FAD) or flavin mononucleotide (FMN), which are derived from the dietary component riboflavin, a water soluble vitamin. Riboflavin regulates the structure and function of flavoenzymes through its cofactors FMN and FAD and, thus, protects the cells from oxidative stress and apoptosis. Hence, it is not surprising that any disturbance in riboflavin metabolism and absorption of this vitamin may have consequences on cellular FAD and FMN levels, resulting in mitochondrial dysfunction by reduced energy levels, leading to riboflavin associated disorders, like cataracts, neurodegenerative and cardiovascular diseases, etc. Furthermore, mutations in either nuclear or mitochondrial DNA encoding for flavoenzymes and flavin transporters significantly contribute to the development of various neurological disorders. Moreover, recent studies have evidenced that riboflavin supplementation remarkably improved the clinical symptoms, as well as the biochemical abnormalities, in patients with neuronopathies, like Brown-Vialetto-Van-Laere syndrome (BVVLS) and Fazio-Londe disease. This review presents an updated outlook on the cellular and molecular mechanisms of neurodegenerative disorders in which riboflavin deficiency leads to dysfunction in mitochondrial energy metabolism, and also highlights the significance of riboflavin supplementation in aforementioned disease conditions. Thus, the outcome of this critical assessment may exemplify a new avenue to enhance the understanding of possible mechanisms in the progression of neurodegenerative diseases and may provide new rational approaches of disease surveillance and treatment.

## 1. Introduction

Energy metabolism generally takes place across the plasma membrane in prokaryotes, whereas eukaryotes have a well-defined specialized organelle called the mitochondrion. Mitochondria are the energy-transducing mobile organelles in eukaryotic cells that produce ATP through the process of oxidative phosphorylation, which drives cellular metabolism [1]. In addition, it acts as a site of various metabolic processes, like the breakdown of sugars and long-chain fatty acids, the synthesis of amino acids, lipids, and steroids, along with numerous other reactions that are essential for the survival of the organism. Mitochondria consist of four components: (i) an outer membrane that has porins, which allows small molecules to enter; (ii) an inner membrane which is impermeable to ions while the transport is mediated by a specific transport system; (iii) cristae; and (iv) the mitochondrial matrix, which contains enzymes involved in the Krebs cycle and electron transport chain (ETC). ETC has evolved to contain the molecular machinery for energy production in the inner mitochondrial membranes, which consists of five protein complexes, among them three of the complexes (I, III, and IV) pump protons (H^+^) to generate a H^+^ gradient for ATP production at complex V.

Mitochondria have their own genome, the mitochondrial DNA (mtDNA), which is located in the mitochondrial matrix. In humans, the mitochondrial genome is a small circular DNA with a size of 16.5 kb [2] that contains 13 polypeptides encoding seven subunits of complex I, one subunit of complex III, three subunits of complex IV, and two subunits of complex V in respiratory chain while genes involved in complex II are encoded by the nuclear DNA. It also contains 22 tRNA and 2 rRNA for its translational mechanism. The biochemistry of mitochondria has been well studied, however, its implications in the development of inborn errors of metabolism have only recently been established and the understanding of mitochondrial diseases caused by the inheritance of genetic variations have gained much significance in recent years. In this review, we discuss the new insights of mitochondrial biology in neurodegenerative diseases.

## 2. Mitochondria—The Power House

The mitochondrial matrix serves as a host for a wide variety of metabolites involved in three major processes, such as citric acid cycle, urea cycle, and electron transport chain. Additionally, mitochondrion contains several electrochemically-active species (flavin adenine dinucleotide (FAD), flavin mononucleotide (FMN), ubiquinone, and cytochrome c4), so it is involved in the production of energy by means of electrochemical mechanisms. The predominant role of mitochondria is the production of ATP, which is known as the energy currency for the proper functioning of the cells. ATP is produced in the cytosol by the oxidation of glucose and pyruvate from dietary food sources by means of cellular respiration (otherwise named as aerobic respiration) [3].

During aerobic respiration, 1%–2% of the consumed oxygen is involved in the production of reactive oxygen species (ROS) that, in excessive amount cause, oxidative damage to DNA and proteins, leads to mitochondrial damage [4]. The continuous production of ROS in mitochondria leads to age-related oxidative stress that result in cellular aging. Furthermore, mtDNA is susceptible to oxidative damage and it is interesting to note that oxidative damage of mtDNA is inversely related to the life span of humans, whereas oxidative damage of nuclear DNA is not related to the life span [5]. Oxidative damage of mtDNA was found to be reduced by reduced glutathione (GSH), however, the oxidation of glutathione increases with ageing in mitochondria of various organs than in whole cells. Thus, it is clear that mitochondria participates in oxidative damage associated with aging.

## 3. Riboflavin in Mitochondrial Pathways

Riboflavin, a water soluble vitamin, acts as a precursor of FMN and FAD, which are involved in key regulatory pathways of mitochondria, such as metabolism of amino acids, fatty acids, and purines, and the oxidation-reduction reaction essential for normal cellular growth and development [5]. Riboflavin consist of an isoalloxazine ring and a ribityl side chain, it is converted to FMN by the addition of phosphate group to the ribityl side chain, and further converted to FAD by the addition of ADP. Enzymes that utilize FMN and FAD are collectively known as flavor coenzymes or flavo proteins.

Riboflavin is considered a vital component of mitochondrial energy production mediated by ETC [6]. It is particularly important for the normal production of ATP, which leads to membrane stability and sustaining adequate energy-related cellular functions. In addition, flavo coenzymes are also involved in drug and toxin metabolism, along with cytochrome P450 [7].

**Figure 1:**
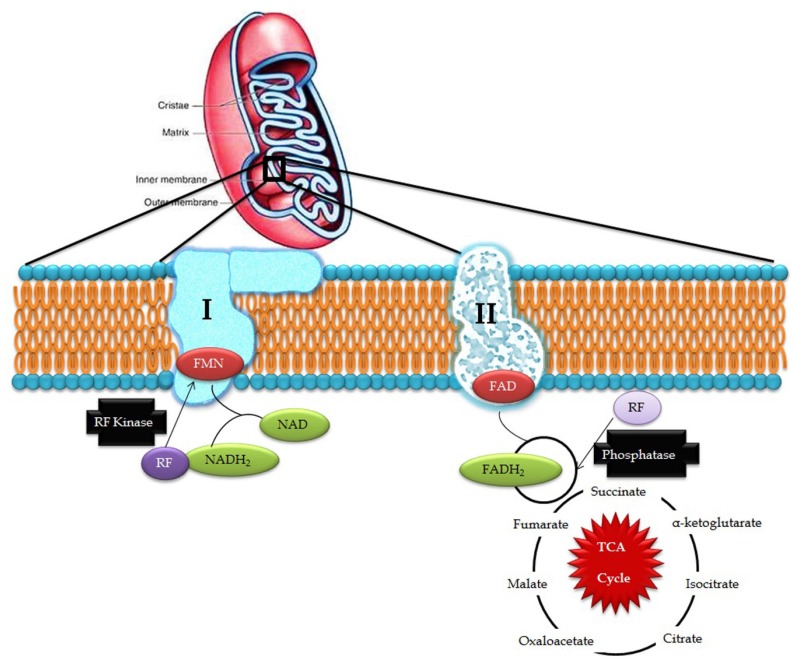
Riboflavin in mitochondrial pathways (RF- riboflavin; FMN- flavin mononucleotide; FAD/FADH_2_- flavin adenine dinucleotide; and NAD/NADH_2_- nicotinamide adenine dinucleotide).

Riboflavin obtained from exogenous dietary sources enters mitochondria from the cytosol by means of specific transporters and is converted to FAD by riboflavin kinase and FAD synthetase which can, be converted to riboflavin by FAD pyrophosphatase. This process is collectively known as the Rf-FAD cycle (Figure 1). In contrast, yeast mitochondria are devoid of FAD synthetase activity, hence, it has to obtain FAD from the cytosol through a specific transporter (FLX1) [8]. Experiments conducted in rat liver mitochondria proved that mitochondria have its own FAD transporter that carries FAD from the cytosol across the mitochondrial membrane for the flavinylation process [9].

In the electron transport chain, FMN acts as a co-factor for NADH-Coenzyme Q reductase which catalyzes the conversion of NADH to CoQ in complex I while FAD is involved in the activity of complex II where it acts as an electron carrier and cofactor for succinate dehydrogenase, which catalyzes the conversion of succinate to fumarate in the Kreb’s cycle and oxidative phosphorylation. Defects in ETC produce free radical superoxide O_2_^−^, hydrogen peroxide, nitric oxide, and highly-reactive hydroxyl radicals, which induce membrane lipid peroxidation and DNA damage. Exposure to excessive reactive oxygen species (ROS) potentially damages nuclear DNA and mtDNA, which is highly deleterious in post-mitotic cells, such as neurons, where cells stop differentiation and thus, cell division is not possible to replace the damaged DNA. Such impairments in the mtDNA lead to bioenergetic dysfunctions that could ultimately cause neuronal cell death [10] and may be involved in the development of mitochondria-associated neurodegenerative disorders [11].

In mitochondrial fatty acid beta oxidation, fatty acids are activated in the cytosol and transported into the inner mitochondrial membrane as carnitine derivatives mediated by carnitine palmitoyl transferase I (CPT I), acylcarnitine translocase (CAT), and carnitine palmitoyl transferase II (CPT II). Within the mitochondrial matrix, the acyl-CoA fatty acids undergo dehydrogenation by acyl-CoA dehydrogenases, which are flavin-dependent and thus, flavins regulate the fatty acid beta oxidation pathway [12].

## 4. Riboflavin Pathogenesis in Mitochondrial Dysfunction

FMN and FAD are chief prosthetic groups that activate various flavoproteins, such as nitric oxide synthase, nitric oxide reductase, and NADPH oxidase, to protect the cell from oxidative stress and apoptosis [13]. Although riboflavin is stored in the liver, spleen, kidney, and cardiac muscle in the form of FAD, and protects these organs against riboflavin deficiency, its half-life is one hour; hence, riboflavin deficiency would impair the proper functioning of these organelles. Furthermore, energy depletion and a decrease in riboflavin kinase activity leads to the insufficient conversion of riboflavin into flavocoenzyme and results in various anomalies, like cataracts, preeclampsia, various types of cancers, and neurological disorders. In addition, deficiency of ETC enzymes, like NADH-CoQ reductase, cytochrome c oxidase, and creatine kinase, leads to infantile mitochondrial myopathy [14].

Recent studies have proved riboflavin supplementation as a therapy to alleviate or reduce the worsening of disease conditions, especially in Brown-Vialetto-Van Laere syndrome (BVVLS) and multiple acyl-CoA dehydrogenase deficiency (MADD) [15]. Although most of the flavo enzymes are encoded by the nuclear genome, surprisingly, they are synthesized and stored in different components of the mitochondria for their active participation in vital pathways, like glycolysis, Kreb’s cycle, beta oxidation, urea cycle, and ETC of mitochondria. When riboflavin is accumulated in the cytoplasm instead of entering into mitochondria, there will be a shortage of riboflavin availability for the flavo coenzymes present in the mitochondria. Hence, riboflavin transporters are essential for the maintenance of riboflavin homeostasis. Till date, only mutations in riboflavin transporters were correlated with neurologically defective phenotypes, while most of the flavoproteins also take part in key regulatory pathways that determine the fate of the cell to undergo either normal or abnormal physiological functions in neurological prospects.

## 5. Riboflavin Related Mitochondrial Dysfunction in Neurological Disorders

**Figure 2:**
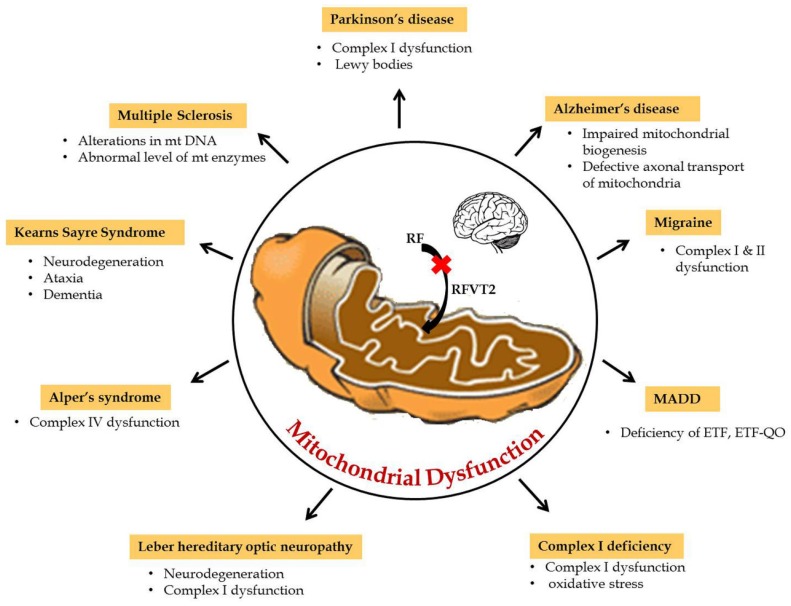
Riboflavin related mitochondrial dysfunction in neurological disorders.

Mitochondria play a key role in the interconnected network to transmit and receive signals where the central nervous system is highly dependent on energy. Furthermore, during neurogenesis for the differentiation and development of axons and dendrites, high amounts of mitochondrial mass is necessary to produce ATP in large quantities [16]. Hence, mitochondrial dysfunction due to any defect in the reduction or oxidative phosphorylation reaction results in impaired oxidative metabolism and diminished energy production which, consequently, leads to neurological disorders. Particularly in amyotrophic lateral sclerosis (ALS), mitochondrial dysfunctions, like abnormal mitochondrial morphology [17], mitochondria-mediated apoptosis [18], and disruption of the axonal transport of mitochondria [19], are the primary reasons for the disease etiology. Riboflavin-related mitochondrial dysfunction in neurological disorders are summarized in Figure 2. In particular, riboflavin deficiency in rats resulted in reduced levels of myelin lipids, cerebrosides, sphingomyelin, and phosphatidylethanolamine in the cerebrum and cerebellum and, consequently, led to the impairment of brain development and maturation [20]. These observations suggested that riboflavin plays a crucial role in the metabolism of essential fatty acids in the brain. Some of the flavo coenzymes involved in mitochondrial dysfunction of neurological diseases are listed in Table 1.

In addition, experiments conducted in chickens showed that riboflavin deficiency resulted in demyelination of peripheral nerve cells. Severity of demyelination was particularly high in Schwann cells, whereas the severity was reduced in spinal nerve roots and distal nerve branches due to nutrient accessibility [21]. Likewise, riboflavin deficiency resulted in the swelling of peripheral nerve trunks and led to peripheral neuropathy in racing pigeons [22]. Henceforth, a detailed knowledge about the role of riboflavin in the mitochondrial dysfunction of neurological disorders is a prerequisite for the discovery of drug targets and treatment.

## 6. Neurological Disorders of Mitochondrial Dysfunction

Disruption of the mitochondrial electron transport chain and other mitochondrial damage leads to several neurological disorders. Some of the riboflavin responsive neurological disorders due to mitochondrial dysfunction are discussed below.

### 6.1. Multiple Acyl-CoA Dehydrogenase Deficiency (OMIM 231680)

Multiple acyl-CoA dehydrogenase deficiency (MADD), also called as glutaric aciduria type II, ethylmalonic-adipic aciduria, and riboflavin-responsive C6-C10 dicarboxylic aciduria, is caused due to the deficiency of the electron transfer flavoprotein (ETF) or its dehydrogenase and ubiquinone oxidoreductase (ETF-QO) [23]. MADD affects various metabolic pathways involving fatty acids and branched amino acids, lysine and tryptophan, and results in a discharge of a variety of distinctive organic acids comprising glutaric, ethylmalonic, 3-hydroxyisovaleric, 2-hydroxyglutaric, 5-hydroxyhexanoic, adipic, suberic, sebacic, and dodecanedioic acids and glycine conjugates due to the impairment of ATP biosynthesis and the accumulation of excessive fatty acids [24]. The blood plasma acylcarnitine pattern also shows distinctive elevation of short-, medium-, and long-chain acylcarnitines ranging from C8 to C16. Features of MADD include muscle weakness, non-epileptic seizures, and atypical migraine with abnormal creatinine level. MADD is diagnosed with mutations in the alpha and beta subunits of electron transfer flavoprotein (*ETFA* and *ETFB*), ETF dehydrogenase (*ETFDH*), FAD synthase (*FADS1*), riboflavin transporters (*SLC52A1-3*), and mitochondrial FAD transporter (*SLC25A32*) [15,25,26,27,28]. Studies have documented that riboflavin supplementation reduced the abnormal behavior and normalized the biochemical profile by regulating the mitochondrial flavo proteome [26,27,29] and are termed as riboflavin-responsive forms of MADD [30].

### 6.2. Brown-Vialetto-Van Laere Syndrome (OMIM 211530)

**Figure 3:**
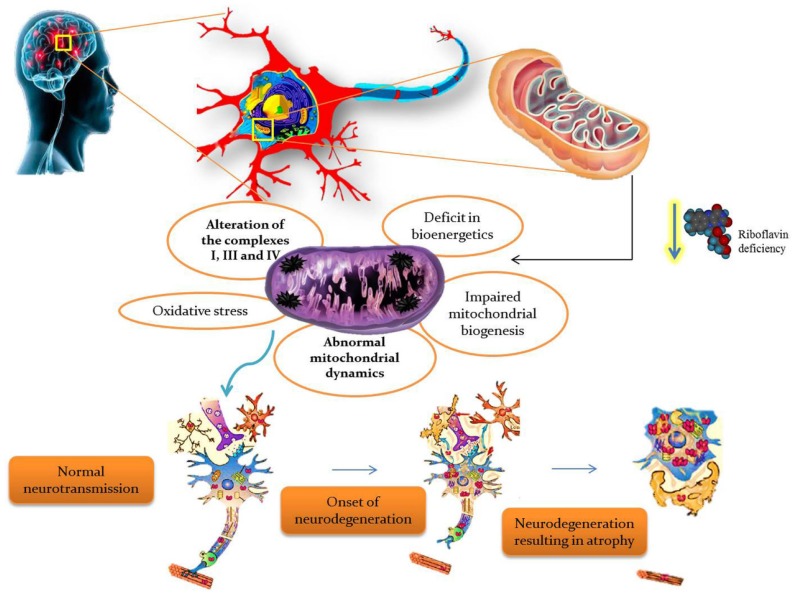
Riboflavin deficiency leading to mitochondrial oxidative stress-mediated neurodegeneration.

BVVLS is a rare, progressive, childhood neurodegenerative disorder characterized by progressive pontobulbar palsy associated with sensorineural deafness. BVVLS has a prominent familial component, consistent with an autosomal-recessive mode of inheritance in most of the patients, while autosomal dominant [31] and X-linked inheritance [32] have also been suggested in a few cases. In BVVLS, bilateral nerve deafness is accompanied by involvement of various motor cranial nerve palsies with VII, IX, and XII, and rarely III, V, and VI, which develop over a relatively short period of time in a previously-healthy individual [32,33]. Generally, BVVLS is clinically heterogeneous, presenting as early as infancy and as late as the third decade of life [33]. Recently, defects in riboflavin transporters *SLC52A3* (formerly C20orf54) [34,35] and *SLC52A2* [35,36] have been identified as the etiology in a large proportion of BVVLS cases. Blood plasma levels of riboflavin and its active coenzyme forms, FAD and FMN, were significantly reduced in BVVLS patients [15]. Moreover, metabolic studies of BVVLS patients revealed the accumulation of acyl-CoA and carnitine esters in the plasma, as well as a urine organic acid profile which both mimic the fatty acid β-oxidation defect seen in patients with MADD. Meanwhile, oral supplementation of riboflavin showed improvement in the clinical symptoms, as well as the biochemical abnormalities in BVVLS patients, signifying that a high dose of riboflavin is a potential treatment for BVVLS [26]. Thus, riboflavin is found to have a critical role in the production of substrates used for the ETC, so it is obvious that any defect in riboflavin transport would impair ETC and consequently lead to neurodegeneration. The overall summary of riboflavin deficiency leading to mitochondrial oxidative stress-mediated neurodegeneration is given in Figure 3.

### 6.3. Complex I Deficiency (OMIM 252010)

The mitochondrial respiratory chain tends to decline with age by affecting complex I and IV of ETC, which leads to mitochondrial myopathies, like cardiomyopathies, encephalomyopathies, and neurological myopathies [37]. Human complex I (NADH-ubiquinone reductase) consists of at least 36 nuclear-encoded and seven mitochondrial-encoded subunits and clinical mutations in any of these subunits are diagnosed to cause this disorder [38]. Functional characterization studies carried out in *Caenorhabditis elegans* with mutation in the active site subunit of complex I revealed that supplementation of riboflavin assembled complex I and reduced oxidative stress, lactic acidosis, and increased metabolic functions [39]. Additionally, riboflavin supplementation normalized the biochemical abnormalities and muscle weakness in an infant with a complex I defect by increasing the cellular availability of FAD [40,41]. Furthermore, mutations in mitochondrial and nuclear genes encoding proteins that are required for proper assembly and stability of the mitochondrial respiratory complex also lead to complex I deficiency. ACAD9 (acyl-CoA dehydrogenase-9), a flavin-dependent acyl carrier, is involved in the proper assembly of complex I through binding with assembly factors NDUFAF-1 and Ecsit [42]. Recently, a missense mutation (Arg532Trp) was diagnosed in the active site of ACAD9 in a Dutch consanguineous family with complex I deficiency (OMIM 611126—complex I deficiency due to ACAD-9), where riboflavin supplementation improved the complex I activity from 17% to 47% in the proband [43].

### 6.4. Leber Hereditary Optic Neuropathy (LHON; OMIM 535000)

LHON is a neurodegenerative disease characterized by acute or subacute loss of central vision and optic atrophy. It arises due to the neurodegeneration of retino-ganglion cells and dysfunction of respiratory chain complex I. Furthermore, it is the first human mtDNA disease identified to be caused by deletion of mtDNA. LHON cases are primarily identified with mutations in any of mitochondrial genes, including MT-ND1, MT-ND4, MT-ND4L, and MT-ND6, and over 95% of cases harbored one of three mtDNA point mutations, G3460A (ND1), G11778A (ND4), and T14484C (ND6), which encodes complex I subunits of the respiratory chain [44]. Studies have documented that supplementation of riboflavin, along with vitamin C and idebenone, in 28 LHON patients reduced the recovery period of dysfunction [45].

### 6.5. Kearns­Sayre Syndrome (OMIM 530000)

Kearns-Sayre Syndrome (KSS) is a rare neuromuscular disorder characterized by ophthalmoplegia, retinitis pigmentosa, chronic inflammation, cortico spinal dysfunction, bulbar palsies, limb girdle muscle weakness, sensory neural hearing loss, progressive neurodegeneration with ataxia, and dementia. Large deletions of mtDNA ranged in size from 2.0 to 7.0 kb [46] are known to cause KSS by the defective oxidative phosphorylation, and the deletions are heteroplasmic. Patients also showed deficiency of cytochome-c oxidase (COX) due to large deletions in the specific region of mtDNA corresponding to the *COX* gene, which was clinically observed as ragged-red fibers in muscle biopsies [47]. Most of the cases are sporadic since mtDNA deletions are inherited very rarely. Deficiency of complex II of the mitochondrial respiratory chain, especially a deficiency of succinic dehydrogenase has been revealed by enzymatic analysis [48]. Since complex II dysfunction is noticed, a combined therapy containing cytochrome c, flavin mononucleotide, and thiamine diphosphate was attempted, which alleviated fatigability, motor disability, corneal edema, and chilblains in the patients, while no improvements were recorded with opthalmoplegia, blepharoptosis, or hearing loss [49]. Recent follow-up study carried out with three complex II-deficient patients showed an improvement in neurological conditions and delayed the early onset. In addition, supplementation of riboflavin to the fibroblast culture showed a two-fold increase in the activities of complex II and succinate dehydrogenase (SDH) [50].

### 6.6. Alper’s Syndrome (OMIM 203700)

Alper’s syndrome is an autosomal recessive disorder characterized by a clinical trial of symptoms, including psychomotor retardation, refractory seizures, and liver failure. It is a mitochondrial DNA depletion disease of the brain that arises due to the degeneration of cerebral gray matter in infancy, characterized by neurodegeneration of basal ganglia. It is caused due to the dysfunction in complex IV of ETC, nuclear-encoded mitochondrial polymerase γ (*PolG1*) deficiency [51], and Twinkle helicase [51,52]. Alper’s syndrome patients with mutations in *POLG* may also undergo complex I deficiency [53]. Since it involves complex I and IV dysfunction, riboflavin could play a possible role in its regulation, which corroborates with the study carried out in *C. elegans* (having mutations in NADH-ubiquinone oxidoreductase) where riboflavin supplementation enhanced the assembly of complex I and IV that further resulted in reduced oxidative stress and increased metabolic functions [39].

### 6.7. Multiple Sclerosis

Multiple sclerosis is an autoimmune disorder that affects the central nervous system through immune cells, potentially also due to alterations in mitochondrial DNA, defective mitochondrial DNA repair mechanisms, abnormal mitochondrial dynamics (fragmentation), impaired trafficking, defective Ca^+^-mediated axonal degeneration, and abnormal levels of mitochondrial enzymes (phosphofructokinase-2 and complex I enzymes) [54]. Earlier, administration of interleukin 6 was found to act against ROS and protect against neuronal cell death, while recent studies carried out in encephalomyelitis C57BL/6 mice showed that riboflavin supplementation reduced the neuronal disability by 26.4% while the use of placebo reduced the risk by 15.4% [55]. Furthermore, riboflavin supplementation leads to a reduction in the expression of BDNF and IL-6 in the brain of an experimental autoimmune encephalomyelitis model of multiple sclerosis, which was correlated with the observed beneficial effects of riboflavin on neurological motor disability and also suggested possible targets of new rational therapeutic strategies for MS [56].

### 6.8. Parkinson’s Disease (OMIM 168600)

Parkinson’s disease (PD) is a progressive movement disorder that is associated with the death of vital nerve cells in the brain. Primary symptoms include tremor, bradykinesia, stiffness of the limbs, and postural instability. It is primarily due to the accumulation of alpha-synuclein protein in the brain as Lewy bodies. In some instances, it is characterized by complex I ETC deficiency where, due to endogenous oxidative damage, the respiratory chain protein complex is affected, which subsequently leads to decreased ATP production, increased free radical production, and results in apoptosis. Deficiency of riboflavin was shown to have impaired oxidative metabolism through reduced glutathione reductase, pyridoxine phosphate oxidase, NADH-ubiquinone reductase, and NADH cytochrome c reductase [57]. Further, supplementation of riboflavin for six months with PD patients showed improvement in the motor capacity from 41% to 71%, and demonstrated to overcome the complex I deficiency. Thus, it is evident that riboflavin could play a role in the conversion of oxidized glutathione to reduced glutathione by catalyzing glutathione reductase, and in the assembly of mitochondrial protein complexes [58]. Recently, a bacterial metabolite produced by *Streptomyces venezuelae* caused dopaminergic neurodegeneration in a PD model of *C. elegans* expressing human α-synuclein due to the impairment of mitochondrial complex I activity. Meanwhile, mitochondrial complex I activators, such as riboflavin and d-β-hydroxybutyrate (DβHB), rescued dopamine neurodegeneration in *C. elegans* by improving both complex I and complex IV activities [59].

### 6.9. Alzheimer’s Disease (OMIM 104300)

Alzheimer’s disease (AD) is a progressive neurodegenerative disorder characterized by short-term memory loss and dementia due to the accumulation of Tau proteins in neurofibrillary tangles, the loss of connection between nerve cells, and extracellular amyloid plaque which leads to mitochondrial fragmentation [60]. Defects in electron transport chain enzymes, such as cytochrome c oxidase (COX) and F(1)F(0)-ATPase, have also been implicated in the progression of AD [61]. Neurodegeneration observed in AD has been suggested to be due to impaired mitochondrial biogenesis, defective axonal transport of mitochondria, and increased DRP1-mediated mitochondrial fission [62]. Hyperhomocysteinemia has been explained as one of the possible mechanisms for neurotoxicity in AD, which is responsible for induced cellular oxidative stress leading to the formation of ROS to cause neuronal cell death. Elevated plasma levels of homocysteine have been documented due to the impaired activity of methylenetetrahydrofolate reductase (MTHFR) in one carbon metabolism of the homocysteine remethylation pathway, which is a FAD-dependent flavoenzyme [63]. Deficiency of cellular FAD has been shown to contribute to the functional impairment of the MTHFR 677T variant genotype and an increase the homocysteine levels, particularly in individuals with low-folate status [64]. Moreover, accumulation of homocysteine for longer periods is thought to be involved in the failure of β-amyloid clearance and damage to the blood brain barrier, which develops into cerebrovascular dysfunction, leading to AD development [65].

## 7. Conclusions

There is a gathering body of evidence which links the interaction between riboflavin and flavoproteins to the protection of neuronal cells from death by oxidative stress and apoptosis. Any anomalous expression and regulation of mtDNA and nDNA encoding of functional proteins in the mitochondria can affect the intracellular levels of FAD and FMN, which are functionally implicated in various pathological conditions leading to neurological disorders. Mitochondrial defects may lead to axonal dysfunction and degeneration through a lack of ATP, increased ROS production, and by modulating the function of various dehydrogenases of the respiratory chain. The understanding of this relationship between mitochondria, rate of neuronal degeneration by oxidative stress, and the protection by riboflavin is at an early stage. Thus, a comprehensive knowledge and new experimental strategies are essential to elucidate the interplay between mitochondrial metabolism, mitochondrial stress, riboflavin transport and metabolism, mtDNA mutation and deletion mechanisms, and the complex neurodegeneration pathways. Such knowledge may provide new targets for combating neurodegenerative diseases.

## Figures and Tables

**Table 1:** Enzymes involved in mitochondrial dysfunction of neurological diseases.

Enzyme	Neurological Disease	Metabolic Function	Location
Succinate dehydrogenase	Complex II deficiency	Krebs cycle	Mitochondrial inner membrane
Acyl Co-A dehydrogenase	Acyl Co-A dehydrogenase deficiency	Beta oxidation	Mitochondrial matrix
Electron transferring flavo protein—Ubiquinone oxidoreductase	Glutamic academia II C	Electron transport chain	Mitochondrial inner membrane
Electron transferring flavo protein	Glutamic academia II A and II B	Electron transport chain	Mitochondrial matrix
NADH - Ubiquinone oxidoreductase	Complex I deficiency	Electron transport chain	Mitochondrial inner membrane
Dihydrolipoyl dehydrogenase, Succinate dehydrogenase and NADH-Ubiquinone oxidoreductase	Leigh Syndrome	Energy metabolism	Mitochondrial matrix
Riboflavin transporter	BVVLS	Riboflavin uptake	Plasma/ Mitochondrial membrane
